# Automatic segmentation of the hippocampus and the amygdala driven by hybrid constraints: Method and validation

**DOI:** 10.1016/j.neuroimage.2009.02.013

**Published:** 2009-07-01

**Authors:** M. Chupin, A. Hammers, R.S.N. Liu, O. Colliot, J. Burdett, E. Bardinet, J.S. Duncan, L. Garnero, L. Lemieux

**Affiliations:** a Department of Clinical and Experimental Epilepsy, Institute of Neurology, UCL, UK; b Cognitive Neuroscience and Brain Imaging Laboratory, CNRS UPR 640, UMPC, Paris France; c Department of Clinical Neuroscience, Division of Neuroscience and Mental Health, Imperial College London

## Abstract

The segmentation from MRI of macroscopically ill-defined and highly variable structures, such as the hippocampus (*Hc*) and the amygdala (*Am*), requires the use of specific constraints. Here, we describe and evaluate a fast fully automatic hybrid segmentation that uses knowledge derived from probabilistic atlases and anatomical landmarks, adapted from a semi-automatic method. The algorithm was designed at the outset for application on images from healthy subjects and patients with hippocampal sclerosis. Probabilistic atlases were built from 16 healthy subjects, registered using SPM5. Local mismatch in the atlas registration step was automatically detected and corrected. Quantitative evaluation with respect to manual segmentations was performed on the 16 young subjects, with a leave-one-out strategy, a mixed cohort of 8 controls and 15 patients with epilepsy with variable degrees of hippocampal sclerosis, and 8 healthy subjects acquired on a 3 T scanner. Seven performance indices were computed, among which error on volumes *RV* and Dice overlap *K*. The method proved to be fast, robust and accurate. For Hc, results with the new method were: 16 young subjects {*RV* = 5%, *K* = 87%}; mixed cohort {*RV* = 8%, *K* = 84%}; 3 T cohort {*RV* = 9%, *K* = 85%}. Results were better than with atlas-based (thresholded probability map) or semi-automatic segmentations. Atlas mismatch detection and correction proved efficient for the most sclerotic Hc. For Am, results were: 16 young controls {*RV* = 7%, *K* = 85%}; mixed cohort {*RV* = 19%, *K* = 78%}; 3 T cohort {*RV* = 10%, *K* = 77%}. Results were better than with the semi-automatic segmentation, and were also better than atlas-based segmentations for the 16 young subjects.

## Introduction

Volumetric analyses of brain structures have become increasingly common, for diagnostic purposes and for identifying disease progression. In this context, the hippocampus (Hc) and the amygdala (Am) are of major importance, due to their implication in epilepsy and Alzheimer's disease. Nevertheless, volume measurement of these structures remains mainly manual, making the study of large cohorts difficult. Fully automatic extraction of Hc and Am is challenging, due to the poor definition of some of their boundaries on Magnetic Resonance Imaging (MRI) scans and consequently prior knowledge (statistical shape information, atlas templates, probabilistic region distributions or anatomical descriptions) has to be taken into account in order to define their boundaries.

Most registration based segmentation methods (Haller et al., 1997 or Dawant et al., 1999), are based on a single subject-template. The subject may be more or less representative of the population, resulting in possible bias (Carmichael et al., 2005, Park et al., 2005), and making intensity refinements necessary (Firbank et al., 2007, Barnes et al., 2008). A more thorough and robust approach (Svarer et al., 2005) would be to use multiple templates, either for building probabilistic information in standard space (Fischl et al., 2002 and 2004; Mega et al., 2005; Pohl et al., 2007; Gouttard et al., 2007), or for information fusion in native (subject) space following parallel registration (Wang et al., 2005 or Heckemann et al., 2006). More complex positional relationship information can also be derived between structures (Fischl et al., 2002), but some of the relationships thus inferred may break down in data from patients, being inferred from specific training samples; to address this specific issue, anatomical knowledge, derived only from anatomical characterisation, has been specifically designed to be stable for controls and patients (Bloch et al., 2005; Barra and Boire, 2001).

Fast fully automatic robust segmentation of healthy and pathological hippocampi and amygdalae suitable for routine use has yet to be demonstrated. On the one hand, segmentations based on global atlas information require high dimensional deformations to be computed, in order to achieve precise extraction (Heckemann et al., 2006). On the other hand, methods based on local anatomical and image information can be fast, but require good initialisation to be efficient and robust with respect to spurious edges (Shen et al., 2002). We have proposed a semi-automatic method using simultaneous region deformation constrained by anatomical landmarks automatically retrieved during the process. Initialisation of the deformation was performed manually by defining a bounding box and placing two seeds, one in the head of the hippocampus and the other in the middle of the amygdala (Chupin et al., 2007).

In this work, we propose an important upgrade of the algorithm that makes it fully automatic and more robust, by incorporating global spatial knowledge in the form of probabilistic atlases. This is based on a probabilistic atlas built in MNI space using MRI data from sixteen healthy subjects and a state-of-the-art registration method (Ashburner and Friston, 2005). Global information can be inferred, which allows to automatically initialise the deformation (bounding boxes and initial objects). The probabilistic information is also used as a new prior in the energy functional, minimised to drive the deformation.

The remainder of the article is organised as follows. First, the algorithm is described, focusing on its novel aspects; second, we evaluate the method by comparing its results with those of manual segmentation, on 1.5 T data from healthy subjects and patients with epilepsy, and on 3 T data from healthy subjects.

## Algorithm description

### Overview of the segmentation algorithm

We present below a global overview of the upgraded operational steps of the method. Briefly, two objects iteratively deform through the reclassification of their border voxels, according to the minimisation of a global energy functional, through an Iterated Conditional Modes (ICM) algorithm. A competitive scheme is used to allow the identification of the partly visible Hc–Am interface. The segmentation is driven by the constraint of anatomical priors, derived from eleven sets of neuroanatomical landmarks. It has been thoroughly described in Chupin et al (2007).

In the new method, global probabilistic information is used to automatically initialise the deformation, namely determine bounding boxes and initial objects. Probabilistic prior knowledge is modelled by low and high likelihood zones, derived from iso-probability regions in the probabilistic atlases, and consisting of voxels with low or high probability of belonging to Hc or Am. This information is then used to constrain the segmentation as described in the section Constraint during the deformations. Furthermore, we have addressed a common problem faced by atlas-based segmentation methods, namely a possible local mismatch of the atlas, which is more frequent in patients with non standard appearance of a structure (here, only Hc). Below is an overview of the whole segmentation process. Only the upgraded steps are described in detail in the following sections (in brackets):

## Atlas construction

### Construction of the Am and Hc probabilistic atlases with healthy controls' datasets) (performed only once)

• Two probabilistic atlases from manually segmented Hc and Am in 16 young healthy subjects.

## Initialisation

### Initialisation of the deformations:

• Registration of the probabilistic atlases for Hc and Am to the subject's native space.
• Automatic definition of a bounding box delimiting the region of interest (ROI, Hc and Am);
• Automatic estimation of intensity characteristics from the ROI intensity histogram;
• Atlas mismatch detection and correction;
• Automatic creation of two initial objects from the maximal probability level for Hc and Am.

## Alternating iterative deformation (as in Chupin et al (2007)

### Constraint during the deformations

• Setting of anatomical landmark set to NULL.

### Homotopic deformation of Hc voxel front

• Selection of re-classification ‘candidates’ in the neighbourhood of the voxels of the Hc front;
• Detection of interface voxels, landmarks and likelihood zones, ICM initialisation;
• Voxel re-classification (ICM energy optimisation): at each iteration, for each voxel candidate, re-classification in the object leading to the smaller local energy:

• For non-interface voxels, re-classification is restricted to either Hc or the background;
• For interface voxels, re-classification is restricted to either Hc or Am.

### Homotopic deformation of Am voxel front

• Selection of ‘candidates’ to re-classification in the neighbourhood of the voxels of the Am front;
• Detection of interface voxels, landmarks and likelihood zones, ICM initialisation;
• Voxel re-classification (ICM energy optimisation):
• For non-interface voxels, re-classification is restricted to either Am or the background;
• For interface voxels, re-classification is restricted to either Am or Hc.

## Convergence criterion checking and termination

### Construction of the Am and Hc probabilistic atlases with healthy controls' datasets

*N* healthy young adult controls (here *N* = 16, S1–S16, age < 35 years), scanned on a 1.5 T Signa scanner (GE Medical Systems, Milwaukee, WI, USA) (see Table 1 for IR-FSPGR sequence parameters), were used to build the probabilistic atlas. The images were manually segmented by an expert following a 3D voxel-based protocol described in Chupin et al (2007). This step resulted in 2*N* = 32 binary-labelled datasets, *N* with right and left Hc {*L*_Hc_^*i*^, *i* = 1...*N*} and *N* with right and left Am {*L*_Am_^*i*^, *i* = 1... *N*}. The transformations from native space to MNI standard space, {*T*^*i*^, *i* = 1...*N*}, were computed using the unified segmentation module available in SPM5 (Ashburner and Friston, 2005, http://www.fil.ion.ucl.ac.uk/spm/software/spm5/), which allows to iteratively optimise simultaneously registration parameters (linear combination of cosine transformation bases), tissue classification, and intensity non-uniformity correction. The registration step was necessary to create a probabilistic atlas, but did not aim at achieving the segmentation only by propagating labels. The default parameters of SPM5 were used.

The binary-labelled datasets were registered to the MNI standard space following *T*^*i*^ (resolution: 0.9375 × 0.9375 × 1.3 in a 256 × 256 × 124 matrix). We obtain the two probabilistic atlases *PA*_Hc_ and *PA*_Am_:

(1) $$\forall v\in \Omega ,\left\{ \begin{array}{l} PA_{\text{Hc}}\left(v\right)=\frac{1}{N}\sum_{i=1}^{N}T^{i}\left(L_{\text{Hc}}^{i}\right)\left(v\right)\\ PA_{\text{Am}}\left(v\right)=\frac{1}{N}\sum_{i=1}^{N}T^{i}\left(L_{\text{Am}}^{i}\right)\left(v\right) \end{array}\right.\text{,}$$

where *v* is a voxel in the MRI set *Ω*. *PA*_Hc_(*v*), respectively *PA*_Am_(*v*), is the probability that *v* belongs to Hc, respectively Am, according to the prior derived from the young controls.

### Initialisation of the deformations

Individual probabilistic atlases, *IPA*_Hc_ and *IPA*_Am_, were created by back-registering the two probabilistic atlases, *PA*_Hc_ and *PA*_Am_, to the subject's space, using the inverse transformation *T^−^*^ *1*^ given by the unified segmentation. If the test dataset was one of the atlas datasets, the atlas was computed with a leave-one-out strategy. Information inferred from the probabilistic atlases was used to automatically determine the limits of two bounding boxes (right, left) and create initial objects for Hc and Am as follows.

#### Bounding boxes

**Fig. 1:**
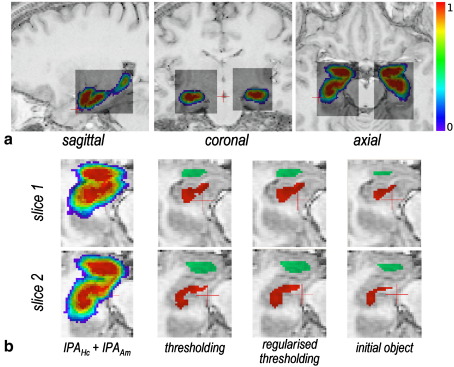
Automatic initialisation illustrated on subject S1. (a) Bounding box extraction, on sagittal, coronal and axial sections. (b) Probabilistic atlas, maximal probability zone (IPA_O_(*v*) = 1) obtained by thresholding and regularised thresholding and initial object, for Hc and Am, on two representative axial slices (one per row).

Bounding box *BB*_HcAm_ extraction mainly aimed at reducing memory load; it should fully contain Hc and Am, but could be larger, as it was not used to geometrically constrain the segmentation. Each bounding box was thus defined as the smallest parallelepiped sub-volumes in *Ω* around the non-null probability Hc*–*Am object HcAm^min^, with an additional layer of one voxel, as illustrated in Fig. 1a.

(2) $${HcAm}^{\text{min}}=\left[v\in \Omega ,IPA_{\text{Hc}}\left(v\right)>0\;\text{or}\;IPA_{\text{Am}}\left(v\right)>0\right]\text{.}$$

#### Initial object

The initial object Hc^init^ (respectively Am^init^), was created from the maximum probability object Hc^max^ (respectively Am^max^). Hc^max^ (respectively Am^max^) was defined as the 1-level of the probability map *IPA*_Hc_ (respectively *IPA*_Am_), which was built by keeping the voxels for which the probability equals one, while regularising to prevent holes (*IPA*_Hc_(*v*)< 1 but *v* is “inside” Hc^max^) and wires (*IPA*_Hc_(*v*) = 1 but *v* “spikes” from Hc^max^) to appear. More precisely, let $N_{O}^{6N}\left(v\right)$ be the number of 6-neighbours of *v* labelled in an object *O*. If $N_{O}^{6N}\left(v\right)$ is larger than 3, rejecting *v* from *O* will result in a hole in *O*; if $N_{O}^{6N}\left(v\right)$ is smaller than 1, including *v* in *O* will result in a wire. Combining regularity rule and probability threshold iteratively, we obtain:

(3) $$\left\{ \begin{array}{l} {\left[{Hc}^{\text{max}}\right]}^{0}=\left[v\in BB_{\text{HcAm}},IPA_{\text{Hc}}\left(v\right)>0\right]\\ {\left[{Hc}^{\text{max}}\right]}^{i}=\left[v\in {\left[{Hc}^{\text{max}}\right]}^{i-1},\begin{array}{c} IPA_{\text{Hc}}\left(v\right)=1\;\text{\&}\;N_{{\left[{Hc}^{\text{max}}\right]}^{i-1}}^{6N}\left(v\right)>1\\ \text{or}\;IPA_{\text{Hc}}\left(v\right)<1\;\text{\&}\;N_{{\left[{Hc}^{\text{max}}\right]}^{i-1}}^{6N}\left(v\right)\geq 3 \end{array}\right] \end{array}\right.\text{,}$$

with equivalent equations for Am^max^. The iterations proceeded until Hc^max^ (respectively Am^max^) remained unchanged. Hc^max^ (respectively Am^max^) was then eroded with a 1 mm-structuring element, and the largest connected component was kept to create the initial objects Hc^init^ (respectively Am^init^). The regularised initial object is illustrated in Fig. 1b.

#### Automatic detection of atlas mismatch and correction strategies

Atlas-based segmentation method can result in errors due to gross mismatches of the registered atlas, most of all in data from patients. Initial tests of the new method showed that some atlas mismatch could occur occasionally. Therefore, a strategy was implemented, based on successive tests comparing intensity characteristics for the grey matter (*GM*) estimated on the bounding box *BB*_HcAm_ and on the initial object Hc^init^ or the 0.5-level probability object Hc^0.5^, defined as:

(4) $${Hc}^{0.5}=\left[v\in BB_{\text{HcAm}},IPA_{\text{Hc}}\left(v\right)\geq 0.5\right]\text{.}$$

The test values were compared with the average and standard deviations for data S1–S16 obtained when using the S1–S16 atlas. Note that the same values were used for all subjects, as no atlas mismatch was detected for subjects S1–S16. The mismatch of the atlas was detected and corrected according to a global to local sequence: detection and correction of a global misplacement of the registered atlas, detection and correction of mis-estimation of atrophy as recovered by the registered atlas, detection and correction of a misplacement of the computed initial objects and, finally, detection of a remaining global misplacement of the atlas. Details on each test are given in Appendix A.

### Constraint during the deformations

The local energy *E*(*v*) was made of five terms for each structure (Chupin et al., 2007): global data attachment (*E*^*G*^), local data attachment (*E*^*L*^), and context terms dedicated to non-stationary anisotropic Markovian regularisation (*E*^*I*^), volume (*E*^*V*^) and surface (*E*^*S*^) control.

(5) $$E_{O}\left(v\right)=\left[E_{O}^{G}\left(v\right)+E_{O}^{L}\left(v\right)+E_{O}^{I}\left(v\right)+E_{O}^{V}\left(v\right)+E_{O}^{S}\left(v\right)\right]\text{.}$$

The regularisation term *E*^*I*^ of the energy functional was modified to take into account the prior probability of the voxel *v* to belong to the deforming object *O* (Hc or Am), derived from *IPA*_Hc_ and *IPA*_Am_. *E*^*I*^ was locally expressed as the comparison of the number of *O*-labelled neighbours of *v*, *N*_*O*_(*v*), and a standard number of neighbours, *Ñ* (13, in 26-connectivity), with a tolerance *σ*^*I*^ around *Ñ*:

(6) $$E_{O}^{I}\left(v\right)={\left(\frac{\tilde{N}-\gamma_{O}^{\mathrm{PZ}}\left(v\right)\gamma_{O}^{\mathrm{AZ}}\left(v\right)\alpha_{O}^{T}\left(v\right).N_{O}\left(v\right)}{\sigma^{I}}\right)}^{5}\text{.}$$

As described in Chupin et al (2007), the α^*T*^ parameter modelled an anisotropic non-stationary behaviour of the regularisation towards the tail. The γ parameters influenced the classification according to prior probabilities of *v* belonging to *O*; for γ values superior to *1*, *N*_*O*_(*v*) was artificially increased, thus decreasing the global energy, and vice versa for values inferior to *1*. The *γ_O_^AZ^* parameter modelled the constraint introduced in anatomical zones *AZ* defined by the anatomical landmarks already described in Chupin et al (2007).

The new *γ_O_^PZ^* parameter modelled the constraint introduced in four probability zones *PZ* inferred from probability levels in *IPA*_Hc_ and *IPA*_Am_. Four levels of constraints (C1–C4), from one *PZ* to four *PZ*, were compared, as detailed in Appendix B.

## Segmentation performance evaluation

Segmentation performance was evaluated based on qualitative and quantitative comparisons between automatic (without and with atlas constraint), semi-automatic (with manual initialisation, as in Chupin et al., 2007) and manual segmentations (Chupin et al., 2007), together with comparison to the 0.5-level object derived from the registered atlas in each subject's space (Hc^0.5^ and Am^0.5^), as a basic atlas-based segmentation.

### Evaluation set and procedure

The method was evaluated on 3 datasets, for which all the acquisition parameters are given in Table 1:

- Group 1: MRI scans of the 16 young controls (age between 20 and 35) (S1–S16 included in Chupin et al., 2007), acquired in the axial plane on a 1.5 T GE scanner, used to create the atlas;
- Group 2: MRI data from 23 subjects from a mixed cohort (Liu et al, 2001), acquired in the coronal plane on another 1.5 T GE scanner; these were split into 3 groups: 8 normal controls (mean age: 40, range: 29–48) (NC1–NC8, Hc volume: 2.9 ± 0.5 cm^3^ (1.8–3.6)), 8 patients with epilepsy and known hippocampal sclerosis (mean age: 42, range: 21–57) (HS1–HS8, 2.0 ± 0.8 cm^3^ (0.7–3.5)), and 7 patients with temporal lobe epilepsy and normal hippocampal volumes (mean age: 37, range: 24–49) (TLE1–TLE7, 2.6 ± 0.5 cm^3^ (1.6–3.4));
- Group 3: MRI scans from 8 further normal controls (mean age: 46, range: 38–53) (NC3T1–NC3T8) scanned on a 3 T GE scanner.

All datasets were manually segmented according to the same protocol as that used to create the probabilistic atlas, by the same investigator. For 3 T data, non-uniformity correction was performed with the unified segmentation (Ashburner and Friston, 2005). All automated segmentations were evaluated by comparison with the reference manual segmentation. A global qualitative evaluation was followed by a quantitative evaluation of the performances, which was based on the quantitative indices described in Chupin et al (2007): relative error in volume *RV*; Dice overlap, *K*, false positives and negatives ratios, *FP* and *FN*; misclassified interface voxels, *MIV*; and two measures based on the symmetric Haussdorf distance: mean value over the surface, *Dm* and maximal value, *DM*. Statistical significance of the differences was evaluated using a paired-samples Student *t*-test with SPSS.

### Implementation issues

Most of the settings described in Chupin et al (2007) for the semi-automatic segmentation were used in this work. Two mechanisms were improved, resulting in different parameters, for intensity estimation and anisotropic regularisation.

Grey matter intensity characteristics are now estimated by fitting three Gaussian curves to the intensity histogram on the ROI (one for the grey matter, one for the white matter and one for the mixture). Average intensity parameters for the global and local data attachment terms described in Chupin et al (2007) are derived from average intensity of grey matter, with predefined ratios as previously (1 for Hc and 0.9 (1.5 T) or 0.95 (3 T) for Am). Tolerances are derived from standard deviation of grey matter intensity, with predefined ratios (1.8 for Hc and 1.1 for Am). The influence of this setting on the segmentation results is discussed in Appendix D.

The anisotropic regularisation in itself is described in Chupin et al (2007), but it is now introduced in a non-stationary way: its weight increases following an anterior–posterior direction in the bounding box *BB*_HcAm_, and the mechanism influences the segmentation more strongly in regions where the tail is anatomically more likely to be present.

New parameters were defined for the atlas constraint, namely the weighting parameter γ*_O_^PZ^*, which was empirically chosen, while keeping it consistent with the γ*_O_^AZ^* values (γ*_O_^AZ^* = 2 for *O*-likely zones and 0.5 for *O*-unlikely zones) (cf Appendix B).

The whole automatic segmentation procedure, including registration, requires less than 15 min for both left and right Hc and Am and is integrated as the SACHA module (Segmentation Automatique Compétitive de l'Hippocampe et de l'Amygdale) in BrainVISA (Cointepas et al., 2001, www.brainvisa.info).

### Validation on the data used for atlas construction (group 1)

**Fig. 2:**
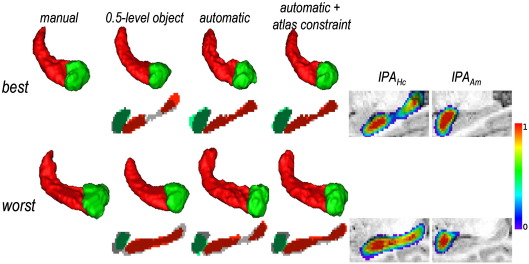
Segmentation results for group 1: 3D-renderings of automatic and manual segmentations, and overlap between segmentations (manual segmentations in shades of grey) and probabilistic atlases on a sagittal slice for the best and worst results (cases S3R and S16R respectively).

The segmentation results were first analysed qualitatively (Fig. 2 for the best and worst results determined as in Chupin et al (2007)). Global visual observation showed that the results were in general more accurate for S1–S16 with the constrained automatic segmentation.

The results were also compared against manual segmentation with the quantitative indices, as shown in Table 2. Overall, the automatic segmentation with the optimal atlas constraint (see Appendix B; “constrained automatic” in Table 2) and the mismatch correction strategy gave accurate results for both Hc and Am. This method performed better than semi-automatic segmentation (Chupin et al., 2007) (for Hc and Am, respectively: improvements of 2 percentage points (*p* = 0.13) and 5 percentage points (*p* = 0.005) for *RV*, 3 percentage points (*p* < 0.001) and 4 percentage points (*p* < 0.001) for *K* and 1 mm (*p* < 0.001) and 0.7 mm (*p* < 0.001) for *DM*). The atlas constraint improved systematically the results (for Hc and Am, respectively: improvements of 2 percentage points (*p* = 0.046) and 6 percentage points (*p* < 0.001) for *RV*, 4 percentage points (*p* < 0.001) and 8 percentage points (*p* < 0.001) for *K* and 1.2 mm (*p* < 0.001) and 1.3 mm (*p* < 0.001) for *DM*) whereas adding the mismatch correction strategy had a negligible effect on the results, due to correct registration. Finally, the automatic segmentation outperformed the atlas-based segmentation given by the 0.5-level object, even if the improvement is not as marked for Am. The last column of the table shows that the anatomical priors are still necessary to ensure accurate segmentation, most of all for the most problematic cases.

### Evaluation on the mixed cohort (group 2)

**Fig. 3:**
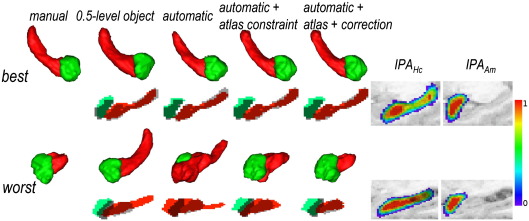
Segmentation results for the patients with Hc sclerosis in group 2: 3D-renderings of automatic and manual segmentations, and overlap between segmentations (manual segmentations in shades of grey) and probabilistic atlases on a sagittal slice for the best and worst results (cases HS8R and HS5L respectively).

Qualitative analyses for controls or non sclerotic structures were comparable to those obtained on the atlas construction cohort. The atlas constraint improved the segmentation, and atlas mismatch was only detected in few cases. Qualitative analysis for sclerotic hippocampi (Fig. 3) revealed the wide shape and signal variation in sclerotic Hc, which made it difficult to predict the registration outcome. In cases with more diffuse sclerosis, the registration succeeded and the segmentation result was quite correct. In cases with more focal sclerosis, the registration was locally inconsistent: atlas mismatch was detected in 6 out of 9 sclerotic Hc and in one normal Hc of a patient with TLE and both Hc of a patient with TLE with grey matter heterotopia in the Hc region. Note the improvement of the segmentation brought by atlas mismatch correction, illustrated for the worst case in Fig. 3.

Quantitative comparisons between automated and manual segmentations are summarised in Table 3. Overall, the automatic segmentation with atlas constraint and the mismatch correction strategy gave correct results for Hc and promising results for Am. This method performed largely better than the semi-automatic segmentation (Chupin et al., 2007) (for Hc and Am, respectively: improvements of 6 percentage points (*p* = 0.018) and 11 percentage points (*p* < 0.001) for *RV*, 3 percentage points (*p* = 0.003) and 3 percentage points (*p* < 0.001) for *K* and 2.7 mm (*p* < 0.001) and 1.1 mm (*p* < 0.001) for *DM*). The atlas constraint systematically improved the results (for Hc and Am, respectively: improvements of 7 percentage points (*p* = 0.009) and 18% (*p* = 0.007) for *RV*, 8 percentage points (*p* < 0.001) and 14% (*p* < 0.001) for *K* and 3 mm (*p* < 0.001) and 2.7 mm (*p* < 0.001) for *DM*); the atlas mismatch correction strategy slightly improved the average results, but was highly useful for sclerotic patients, as described in the following paragraph. Finally, the automatic segmentation outperformed the atlas-based segmentation given by the 0.5-level object, even if the improvement is not obvious for Am; note that the potentially pathological structure was only Hc. As for controls, the anatomical priors are still necessary to ensure accurate segmentation, most of all for the most problematic cases.

Results for the fully automatic segmentation with atlas constraint and mismatch correction were *RV* = 14%, *K* = 77% and *DM* = 5 mm for the 9 sclerotic Hc. The greater shape and signal variations in these Hc is again shown by the overall inadequacy of the 0.5-level object (*RV* = 39%, *K* = 59% and *DM* = 6.4 mm for the 9 sclerotic Hc). The semi-automatic segmentation was unable to cope with the loss of image quality in the hippocampal region in some cases (*RV* = 31%, *K* = 73% and *DM* = 10.3 mm for the 9 sclerotic Hc). Finally, the effect of the mismatch correction on the quantitative values for Hc was large for some subjects, leading to an improvement of 19 percentage points for the maximal *RV* value and 11 percentage points for the minimal *K* value overall sclerotic Hc, indicating that the correction was efficient for cases for which the segmentation failed otherwise. Note that, after correction, the maximal value for *RV* (35%) was obtained for HS3L, for which the manually estimated volume was the smallest in the group studied, namely 0.7 cm^3^; the error corresponds to 0.3 cm^3^ or 10% of the average manual volume of NC1–NC8.

### Evaluation on the 3T control cohort (group 3)

**Fig. 4:**
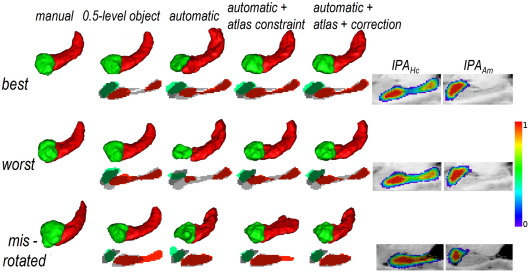
Segmentation results for group 3: 3D-renderings of automatic and manual segmentations, and overlap between segmentations (manual segmentations in shades of grey) and probabilistic atlases on a sagittal slice for the best and worst results (cases NC3T2L and NC3T4L respectively) and NC3T7L, for which atlas mismatch is detected.

The segmentation was qualitatively correct for all the subjects, as illustrated in Fig. 4 top rows. For one subject (NC3T7) the left Hc appeared mis-rotated, resulting in a mismatch of the registered atlas, correctly detected and corrected by the automatic strategy, as illustrated in Fig. 4 bottom row.

The results of the quantitative analysis are given in Table 4. Overall, the automatic segmentation with the atlas constraint and the mismatch correction strategy gave accurate results for Hc and promising results for Am. This method performed better than the semi-automatic segmentation (Chupin et al., 2007) (for Hc and Am, respectively: improvements of 0 percentage points (*p* = 0.85) and 2 percentage points (*p* = 0.63) for *RV*, 2 percentage points (*p* = 0.013) and 4 percentage points (*p* = 0.008) for *K* and 0.4 mm (*p* = 0.047) and 1 mm (*p* = 0.001) for *DM*). The atlas constraint systematically improved the results (for Hc and Am, respectively: improvements of 6 percentage points (*p* = 0.002) and 16 percentage points (*p* = 0.006) for *RV*, 6 percentage points (*p* = 0.001) and 15 percentage points (*p* < 0.001) for *K* and 2.4 mm (*p* = 0.023) and 2.1 mm (*p* < 0.001) for *DM*). Mismatch was detected only for the left Hc of NC3 T7 and the correction resulted in an improvement for *K* from 66 to 78%, and for *DM* from 11.5 mm to 4.8 mm. Finally, the automatic segmentation outperformed the atlas-based segmentation given by the 0.5-level object for Hc, but the tendency was reversed for Am; this may be due to decrease in contrast in these datasets and lower variability in Am than in Hc. As for controls, the anatomical priors are still necessary to ensure accurate segmentation, most of all for the most problematic cases.

## Discussion

We have demonstrated that the introduction of hybrid knowledge, in the form of local anatomical priors and global and local spatial probabilistic priors, allows robust accurate, fast fully automatic segmentation of the hippocampus and the amygdala. In addition to being fully automatic, the new method is more robust than the semi-automatic one to variation in image quality and image acquisition parameters. In patients with hippocampal sclerosis, the segmentation was made more difficult by the wide variety of sclerosis patterns and poorer image quality. Nevertheless, the full method which incorporates a simple strategy for automatic detection and correction of local atlas mismatch resulted in consistent segmentations even for patients with severe sclerosis.

A key point of the method is that both control and patient datasets are segmented with the same parameters and the same atlas, built with datasets from young healthy controls. Robustness with respect to acquisition parameters is greatly increased in the new method compared to the previous, semi-automatic, version (Chupin et al., 2007). We have also shown that both the atlas and anatomical constraints benefit the segmentation, as accuracy decreases when the anatomical constraint is removed. The segmentation performance is stable in relation to relatively important variations in parameter settings, introduced in data attachment energy terms and initialisation, as shown in Appendix D, and related to atlas constraint, as shown in Appendix B and C. It has also been tested on other datasets, as detailed in Chupin et al (2008).

Regarding atlas registration methods, exact registration is not necessary, as the segmentation is not directly derived from the atlas. On the one hand, semi-deformable registration was preferred to a rigid transformation, as it gave the means to encompass larger variability and improve the probabilistic constraint, while remaining fast. SPM5's registration method was chosen due to its availability and rapidity, and considering that more deformable methods, such as the one described in Heckemann et al (2006), would not necessarily have resulted in a perfect match, as demonstrated in Hammers et al (2007) where a correction of the registered atlases by the SPM5 grey matter map was used for patients with epilepsy. On the other hand, it was not necessary to use a fully-deformable registration method, which would have been more time consuming, as for example the LDDMM method used in Khan et al (2008), which needs around 90 min for each structure on a subvolume, with parallel computing.

For the hippocampus, the performance of the fully automatic method was superior to that of both semi-automatic and atlas-based (derived from the 0.5-level object) versions. The preliminary results of the automatic strategy for detection and correction of initial mismatch allowed more robust segmentation of extreme cases. More extensive tests in other populations are required to ensure that it is robust enough for a large range of acquisition sequences and pathological hippocampi. Nevertheless, most of the mismatches which are detected are located at the interface with the ventricle, which makes the detection more robust to variations in the image acquisition parameters than if they were between white and grey matter. The mismatch correction strategy remains very simple and more complex strategies, for example including rotations and a stochastic search in the neighbouring positions, will be evaluated.

For the amygdala, the comparison of the performances of the different methods is not so straightforward: except for the young controls S1–S16, the results of the atlas-based segmentation are comparable or better than the results of the automatic segmentation. This can be explained by a lower variability for the amygdala compared to the hippocampus in our samples, no amygdala-specific pathology being considered, and also by less well-defined borders on MRI for the amygdala, particularly for the anterior border, which is not clearly visible on MR images and makes the refinement step less robust.

### Comparison with other approaches

Published results for segmentation methods are difficult to compare because of different subject samples, image quality and evaluation strategies. Nevertheless, a comparison of values reported in the literature gives a rough estimate of the relative performance of our segmentation method. For healthy controls, the segmentation results (a *K* overlap value of 87% for Hc and 84% for Am) compare favourably with the published studies for which a quantitative evaluation is available. Furthermore, few methods are evaluated on both 1.5 T and 3 T data. Other segmentation methods have been proposed in the literature, using a first step based on the registration of a probabilistic atlas and a intensity refinement step (Firbank et al., 2007, and Barnes et al., 2008); nevertheless, these methods do not include any mechanism to ensure 3D coherence and register single subject templates. Most importantly, our segmentation is less biased, because the atlas-derived constraint is not binary, and its effect is balanced by the anatomical priors and the data attachment terms; this is demonstrated by the behaviour of the segmentation results while varying the atlas definition. Results for the learning curve and using an atlas built with a very different manual segmentation protocol are given in Appendix C. Furthermore, the segmentation is more robust than the above mentioned methods, because our homotopic and regularised segmentation mechanism ensures 3D coherence of the segmented object.

Amongst single atlas-based methods, *K* is 84% and *RV* is 6% for Hc (Hogan et al., 2000) after the manual placement of 28 landmarks, with the HDBM method described by Haller et al (1997), registering a single subject atlas; the method is also initialised by the FreeSurfer segmentation in Khan et al (2008), to make it fully automatic, but the *K* values are only 75% for Hc in young healthy subjects. Recently, a method based on the registration and segmentation module of SPM5, using a Hc template drawn in MNI space (Firbank et al., 2007) was evaluated on 9 elderly controls with an *RV* of 5% and a *K* of 74%.

Amongst multiple or probabilistic atlases approaches, a high dimensional deformation of a probabilistic atlas followed by a thresholding of the probability maps (Gouttard et al., 2007), gives *K* value of around 70% for both Hc and Am; note that there may be some differences in the protocol used to build the atlas and that used for the test subjects. Heckemann et al (2006) registered independently pre-segmented subjects to a target, with a high-dimensional deformation method followed by determining the most probable label, and report *K* values of 82% for Hc and 81% for Am. For subjects with medial temporal lobe epilepsy, the same method combined with grey matter thresholding based on SPM5 yielded *K* values of 83% for non-sclerotic Hc (Hammers et al., 2007). Another method, based on finding the best match amongst a library of Hc templates, with a refinement step based on intensity thresholds and conditional dilation, was evaluated on 19 elderly controls, with a *K* of 84% (Barnes et al., 2008).

Some hybrid methods were also reported, using atlas-based approaches and classification, as in the approach we propose. The method developed by Fischl et al. (2002) for FreeSurfer, based on a Markovian classification using priors derived for a probabilistic atlas, achieved 80%/65% for Hc / Am. In a more recent study (Han and Fischl, 2007), the algorithm was made less sensitive to scanner platform, by renormalizing atlas intensity distributions; it was tested on two samples acquired on two different scanners, with *K* values for Hc/Am of 79% / 69%, on the new scanner and 86% / 81%, on the “training” scanner, respectively. Pohl et al (2007) reported the introduction of a probabilistic atlas prior in a hierarchical framework, and *K* values were 81% for Hc and 86% for Am.

Our results on patient data also compare favourably with published results in patients with epilepsy. Few methods have been thoroughly evaluated in patient data. Values for the 9 sclerotic Hc in HS1–8 (volume: 1.4 cm^3^ (0.7–2 cm^3^), average volume for normal controls: 2.9 cm^3^) are *K* = 77% (68–85) and *RV* = 14% (4–35), while in Hogan et al (2000), they were, for 5 sclerotic Hc (volume: 1.3 cm^3^ (1.2–1.4 cm^3^), average volume for healthy side: 2.8 cm^3^), *K* = 66% (57–75) and *RV* = 16% (6–19). A recent proof-of-principle study in 9 sclerotic Hc (Hammers et al., 2007) (volume: 1.3 cm^3^ (1.1–1.6 cm^3^), average volume for normal controls: 2.4 cm^3^) achieved similar overlap (*K* = 76% (71–83)) but at the expense of ∼ 360 h of CPU time (or ∼ 12 h, if parallelised) versus 15 min (including ∼ 10 min for SPM5 registration) for the method presented here.

## Conclusion

In conclusion, the method we proposed proved fast, accurate, robust to changes in acquisition parameters and field strength, and quite robust to pathology. The method has been validated on data from different institutions and patients with epilepsy with known hippocampal sclerosis. One of its main advantages is to require only one single probabilistic atlas for segmenting even highly pathological structures, as the method uses the information derived from probabilistic atlases in combination with anatomical priors. Results of the automatic strategy for detection and correction of mismatches of the registered atlas proved efficient in cases when it was most necessary, namely patients with focal sclerosis.

## Figures and Tables

**Fig. 5:**
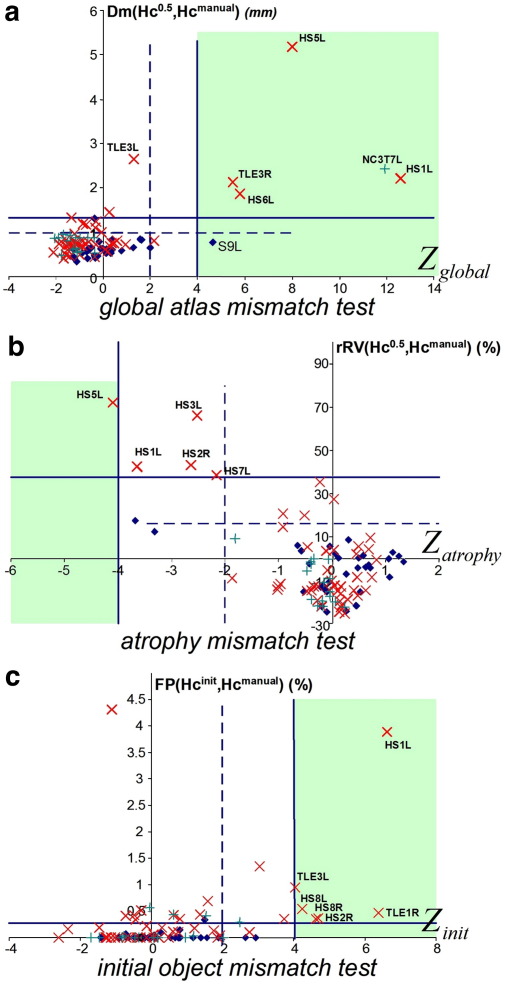
Measure of actual atlas mismatch (*Y*-axis) as a function of detection tests (*X*-axis: *z*-score of the test value with respect to S1–S16): (a) global mismatch (measure = mean symmetric distance between the 0.5-level object and the manual segmentation); (b) atrophy mismatch (measure = relative error on volumes between the 0.5-level object and the manual segmentation); (c) initial object mismatch (measure = false positives ratio between the initial object and the manual segmentation). The solid vertical line indicates the threshold value and the dashed line indicates the median between the average value and the threshold. All the points in the green area are those for which mismatch is detected.

**Fig. 6:**
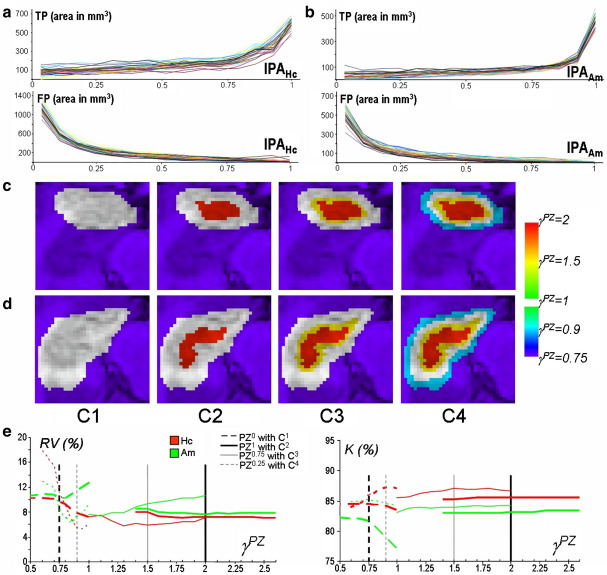
Definition of the probabilistic atlas constraints C1, C2, C3, and C4. (a and b) Histogram of the area corresponding to voxels which are inside (true positives, *TP*) and outside (false positives, *FP*) the manual segmentation as a function of IPA_O_(v), for the leave-one-out atlases, for S1–S16 (a. for Hc and b. for Am). (c and d) probability zones (*PZ*) used in the 4 levels of probabilistic atlas constraint (C1-C4) evaluated for the method, on an axial slice (c. for Am and d. for Hc). (e) Sensitivity to γ^PZ^ parameters (*RV* and *K* for Hc (in red) and Am (in green) when varying γ^PZ^) (see text for details). (For interpretation of the references to colour in this figure legend, the reader is referred to the web version of this article.)

**Table 5:** Quantitative indices for the segmentation run with the 4 levels of probabilistic atlas constraint for S1–S16

object	Index	*C*1	*C*2	*C*3	*C*4
Hc	*RV* (%)	10 ± 10 (0–40)	7 ± 6 (1–20)	6 ± 4 (0–13)	5 ± 4 (0–16)
*K* (%)	85 ± 4 (74–91)	85 ± 3 (80–91)	87 ± 3 (82–93)	87 ± 3 (81–93)
*MIV* (%)	0.4 ± 0.5 (0–2)	0.5 ± 0.5 (0–2)	0.6 ± 0.7 (0–2.8)	0.6 ± 0.7 (0–3.3)
*DM* (mm)	4.6 ± 1.9 (1.9–8.8)	4 ± 1.3 (2.1–6.6)	3.6 ± 1 (2.3–5.9)	3.5 ± 1.1 (1.9–7.3)
Am	*RV* (%)	10 ± 8 (0–32)	8 ± 7 (0–31)	9 ± 6 (0–26)	7 ± 6 (1–29)
*K* (%)	82 ± 4 (73–91)	83 ± 4 (75–91)	84 ± 4 (75–91)	85 ± 4 (75–92)
*MIV* (%)	1.2 ± 0.8 (0.2–3.6)	1.1 ± 0.8 (0–3.6)	1.2 ± 1.1 (0.1–5.0)	1.1 ± 1 (0.1–4.0)
*DM* (mm)	3.6 ± 0.6 (2.1–4.8)	3.3 ± 0.6 (1.9–5.0)	3.2 ± 0.6 (1.9–5.0)	3.2 ± 0.6 (1.6–5.0)

Values = average ± standard-deviation (minimum– maximum).

**Table 6:** Quantitative indices for atlases derived from various numbers of subjects and from another set of subjects and manual segmentations (IBSR) for a. the atlas-constrained automatic segmentation and b. the 0.5-level object for S1–S16.

	Index	4	8	12	15	16	*IBSR* 10
*a. Atlas constrained automatic segmentation*							
Hc	*RV* (%)	8 ± 7 (1–27)	7 ± 6 (0–23)	6 ± 5 (0–19)	5 ± 4 (0–16)	5 ± 5 (0–18)	7 ± 5 (1–19)
*K* (%)	85 ± 3 (79–90)	86 ± 3 (78–91)	87 ± 3 (79–93)	87 ± 3 (81–93)	88 ± 3 (80–94)	85 ± 3 (76–89)
*MIV* (%)	0.6 ± 0.7 (0–3.3)	0.7 ± 0.9 (0–4.3)	0.6 ± 0.9 (0–4.4)	0.6 ± 0.8 (0–3.3)	0.6 ± 0.7 (0–2.4)	0.9 ± 0.8 (0–3)
*DM* (mm)	4.2 ± 2.2 (2–14)	4 ± 2.2 (1.9–13)	3.5 ± 1 (2.3–7.3)	3.5 ± 1.1 (1.9–7.3)	3.7 ± 1.6 (1.9–11)	4 ± 1.3 (2–8)
Am	*RV* (%)	10 ± 8 (0–35)	8 ± 7 (0–33)	7 ± 6 (1–30)	7 ± 6 (1–29)	7 ± 6 (0–28)	11 ± 9 (1–34)
*K* (%)	84 ± 4 (71–91)	85 ± 4 (75–92)	84 ± 4 (76–92)	85 ± 4 (75–92)	85 ± 4 (75–92)	81 ± 4 (74–89)
*MIV* (%)	1.7 ± 2.1 (0–10)	1.3 ± 1.4 (0–6.5)	1.1 ± 0.9 (0.1–3.5)	1.1 ± 1 (0.1–4.0)	1.1 ± 0.8 (0.1–3.3)	0.7 ± 0.6 (0–2.2)
*DM* (mm)	3.1 ± 0.6 (2.1–4.8)	3.2 ± 0.6 (1.9–4.9)	3.2 ± 0.6 (1.6–5.0)	3.2 ± 0.6 (1.6–5.0)	3.2 ± 0.6 (1.6–5.0)	3.8 ± 0.8 (2.6–5.6)

*b. 0.5-Level object*							
Hc	*RV* (%)	17 ± 9 (2–40)	16 ± 8 (2–32)	11 ± 8 (0–27)	10 ± 6 (1–24)	9 ± 8 (0–25)	24 ± 11 (4–49)
*K* (%)	73 ± 5 (58–81)	74 ± 5 (60–83)	77 ± 4 (62–85)	78 ± 5 (64–85)	80 ± 5 (66–87)	74 ± 4 (64–80)
*MIV* (%)	1.2 ± 1.2 (0–6)	1 ± 1.3 (0–6)	0.8 ± 1.1 (0–5.4)	0.8 ± 1.1 (0–5)	0.7 ± 0.9 (0–4.5)	3.1 ± 1.8 (0.2–7.6)
*DM* (mm)	4.7 ± 1.4 (3.1–10)	4.5 ± 1.2 (2.9–8.9)	4.2 ± 1.3 (2.8–8.6)	4 ± 1.3 (2.6–8.5)	3.9 ± 1.3 (2.3–8.9)	9.1 ± 2 (5.2–13)
Am	*RV* (%)	15 ± 11 (0.–34)	15 ± 9 (1–40)	12 ± 10 (0–40)	10 ± 8 (0–33)	10 ± 8 (1–36)	15 ± 9 (2–42)
*K* (%)	80 ± 5 (66–88)	81 ± 5 (67–88)	82 ± 4 (69–90)	83 ± 4 (70–89)	84 ± 4 (72–91)	76 ± 4 (68–82)
*MIV* (%)	2.2 ± 2.6 (0–11.1)	2.2 ± 2.7 (0–10.7)	2.1 ± 2.4 (0–9.4)	2.1 ± 2.4 (0–9.3)	1.8 ± 2.2 (0–8.3)	0.8 ± 1.1 (0–4.3)
*DM* (mm)	3.1 ± 0.7 (2.3–4.8)	2.9 ± 0.5 (1.9–4.3)	2.9 ± 0.6 (2.1–4.8)	2.8 ± 0.5 (1.9–4.0)	2.7 ± 0.6 (1.9–4.0)	3.8 ± 0.8 (3–5.7)

Values = average ± standard-deviation (minimum – maximum).

**Fig. 7:**
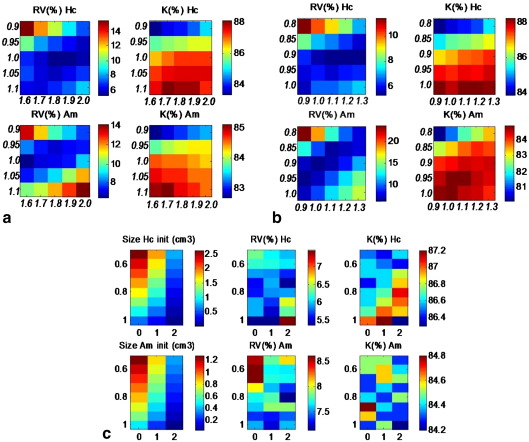
Quantitative indices for S1–S16 with the leave-one-out atlas when varying parameters. a and b when varying ratios controlling intensity parameters with respect to grey matter intensity estimate. (a) the ratios for average intensity (*Y*-axis) and tolerance (*X*-axis) are let varying for Hc, respectively between 0.9 and 1.1 (default = 1.0) and 1.6 and 2 (default 1.8). (b) the ratios for average intensity (*Y*-axis) and tolerance (*X*-axis) are let varying for Am, respectively between 0.8 and 1.0 (default = 0.9) and 0.9 and 1.3 (default 1.1). (c) when varying initialisation parameters for both structures at the same time (*Y*-axis: probability between 0.5 and 1 (default = 1); *X*-axis: erosion structuring element between 0 and 2 (default = 1)).

**Table 1:** Acquisition parameters for the MRI datasets used to build the atlas and in the evaluation process.

	TR	TE	TI	Flip angle	Slice thickness	Voxel size	Orientation	Matrix	NEX
group 1	14.3 ms^a^	6.3 ms^a^	600 ms	10°	1.3 mm^b^	0.9375 mm	axial	256 × 192^c^	1
group 2	17.4 ms	4.2 ms	450 ms	20°	1.5 mm	0.9375 mm	coronal	256 × 192	1
group 3	8.2 ms	3.2 ms	450 ms	20°	1.1 mm	0.9375 mm	coronal	256 × 256	1

a TR = 10.5 ms and TE = 2.2 ms for S15, TR = 10.3 ms and TE = 2.1 ms for S9.

b Slice thickness of 1.5 mm for S7–S9.

c 256 × 256 matrix for S15.

**Table 2:** Quantitative indices for comparison against manual segmentation for group 1 (average value ± standard deviation (minimum – maximum)).

	Index	Semi-automatic	0.5-Level object	Automatic	Constrained automatic	Corrected automatic	Constrained automatic no anatomical prior
Hc	*RV* (%)	7 ± 4 (0–14)	10 ± 6 (0–26)	7 ± 6 (0–27)	5 ± 4 (0–16)	5 ± 4 (0–16)	12 ± 8 (1–28)
*K* (%)	84 ± 3 (78–89)	80 ± 5 (65–86)	83 ± 4 (76–90)	87 ± 3 (81–93)	87 ± 3 (81–93)	83 ± 5 (69–88)
*FP* (%)	15 ± 3 (10–20)	18 ± 6 (7–36)	14 ± 4 (6–22)	11 ± 3 (5–18)	11 ± 3 (5–18)	19 ± 6 (9–33)
*FN* (%)	13 ± 5 (4–21)	15 ± 4 (6–24)	14 ± 6 (6–29)	12 ± 4 (6–22)	12 ± 4 (6–21)	10 ± 4 (5–21)
*MIV* (%)	1.1 ± 1 (0–3.7)	0.8 ± 1 (0–4.7)	1.4 ± 2 (0–7.8)	0.6 ± 0.8 (0–3.3)	0.6 ± 0.7 (0–3.3)	0.8 ± 0.7 (0–3.2)
*Dm* (mm)	0.5 ± 0.1 (0.4–.8)	0.6 ± 0.2 (0.4–1.4)	0.5 ± 0.2 (0.3–0.9)	0.4 ± 0.1 (0.2–0.6)	0.4 ± 0.1 (0.2–0.6)	0.6 ± 0.3 (0.3–1.5)
*DM* (mm)	4.5 ± 1.5 (2.5–9)	3.8 ± 1.2 (2.6–.5)	4.7 ± 1.5 (2.6–7.9)	3.5 ± 1.1 (1.9–.3)	3.5 ± 1.1 (1.9–7.3)	4.3 ± 1.8 (2.8–9.8)
Am	*RV* (%)	12 ± 7 (1–27)	10 ± 7 (0–29)	13 ± 8 (1–30)	7 ± 6 (1–29)	7 ± 6 (1–29)	13 ± 7 (0–26)
*K* (%)	81 ± 4 (69–88)	84 ± 4 (71–90)	77 ± 6 (62–88)	85 ± 4 (75–92)	85 ± 4 (76–92)	83 ± 4 (72–91)
*FP* (%)	19 ± 6 (6–32)	14 ± 6 (3.9–28)	20 ± 8 (6–34)	14 ± 5 (4–26)	14 ± 5 (4–26)	19 ± 6 (5–28)
*FN* (%)	13 ± 5 (5–25)	13 ± 6 (4–30)	17 ± 7 (6–30)	12 ± 4.4 (6–28)	12 ± 4.3 (6–28)	10 ± 4 (5–23)
*MIV* (%)	1.5 ± 1 (0.3–3.8)	2 ± 2.3 (0–8.9)	1.2 ± 0.8 (0–3.8)	1.1 ± 1 (0.1–4.0)	1.1 ± 1 (0.1–4.0)	1.4 ± 1.7 (0–6.5)
*Dm* (mm)	0.7 ± 0.2 (0.4–1.2)	0.5 ± 0.1 (0.3–.9)	0.8 ± 0.3 (0.4–1.4)	0.5 ± 0.1 (0.2–1.0)	0.5 ± 0.1 (0.2–1.0)	0.6 ± 0.2 (0.3–0.9)
*DM* (mm)	3.9 ± 0.9 (2.8–6)	2.7 ± 0.5 (1.6–7.9)	4.5 ± 1.1 (2.8–7.3)	3.2 ± 0.6 (1.6–5.0)	3.2 ± 0.6 (1.6–5.0)	3.5 ± 0.6 (1.9–5)

Values = average ± standard-deviation (minimum – maximum).

**Table 3:** Quantitative indices for comparison against manual segmentation for the group 2 (average value ± standard deviation (minimum – maximum)).

	Index	Semi-automatic	0.5-Level object	Automatic	Constrained automatic	Corrected automatic	Corrected automatic no anatomical prior
Hc	*RV* (%)	14 ± 18 (0–114)	18 ± 15 (1–72)	15 ± 19 (0–109)	9 ± 10 (0–54)	8 ± 7 (0–35)	23 ± 22 (1–98)
*K* (%)	81 ± 8 (35–88)	73 ± 11 (42–86)	76 ± 10 (40–87)	83 ± 8 (57–90)	84 ± 5 (68–90)	76 ± 15 (15–87)
*FP* (%)	15 ± 7 (3–37)	20 ± 13 (6–59)	22 ± 13 (5–72)	15 ± 9 (7–46)	14 ± 6 (7–33)	27 ± 14 (10–68)
*FN* (%)	17 ± 11 (3–74)	22 ± 7 (8–39)	16 ± 6 (4–36)	14 ± 6 (5–36)	14 ± 5 (5–33)	10 ± 6 (2–29)
*MIV* (%)	2.3 ± 4 (0–21)	2.3 ± 4.6 (0–28)	10 ± 13 (0–49)	3 ± 7.2 (0–44)	2.6 ± 4.8 (0–27)	2.5 ± 4.9 (0–24)
*Dm* (mm)	0.9 ± 1.1 (0.4–.8)	1 ± 0.8 (0.4–5.2)	1.1 ± 1 (0.4–6.2)	0.6 ± 0.5 (0.3–2.5)	0.5 ± 0.3 (0.3–.5)	1.1 ± 1.4 (0.4–8)
*DM* (mm)	6.8 ± 3.8 (2.4–23)	4.5 ± 2.9 (2.1–20)	7.1 ± 3.2 (3.1–18)	4.4 ± 2.2 (2.6–12)	4.1 ± 1.6 (2.6–9)	5.5 ± 3.7 (2.6–20)
Am	*RV* (%)	30 ± 18 (1–70)	15 ± 10 (0–45)	37 ± 44 (0–200)	19 ± 13 (0–56)	19 ± 12 (1–56)	22 ± 15 (1–62)
*K* (%)	75 ± 6 (60–82)	75 ± 8 (36–86)	64 ± 19 (0–86)	78 ± 8 (38–89)	78 ± 6 (53–89)	77 ± 8 (46–89)
*FP* (%)	31 ± 10 (9–53)	18 ± 9 (3–36)	24 ± 11 (0–50)	23 ± 9 (7–45)	23 ± 9 (7–45)	25 ± 10 (10–48)
*FN* (%)	10 ± 8 (1–37)	21 ± 9 (8–45)	27 ± 24 (1–100)	13 ± 9 (2–47)	13 ± 8 (2–43)	11 ± 8 (2–46)
*MIV* (%)	3.9 ± 4 (0.1–18.8)	2.9 ± 3.4 (0–13.2)	1.7 ± 2.7 (0–14.4)	2.1 ± 2.7 (0–11.6)	2 ± 2.7 (0–11.6)	3 ± 4.7 (0–26)
*Dm* (mm)	1 ± 0.3 (0.5–1.7)	0.8 ± 0.3 (0.4–.9)	1.8 ± 2.1 (0.5–10)	0.8 ± 0.3 (0.3–1.8)	0.7 ± 0.3 (0.3–1.6)	0.8 ± 0.3 (0.3–1.9)
*DM* (mm)	4.7 ± 1.2 (3.1–8.5)	3.5 ± 1 (2–7.3)	6.3 ± 3.3 (3.2–17)	3.6 ± 0.9 (2–6.9)	3.6 ± 0.9 (2–6.9)	3.6 ± 0.9 (2–6.0)

Values = average ± standard-deviation (minimum – maximum).

**Table 4:** Quantitative indices for comparison against manual segmentation for group 3 (average value ± standard deviation (minimum – maximum)).

	Index	Semi-automatic	0.5-Level object	Automatic	Constrained automatic	Corrected automatic	Corrected automatic no anatomical prior
Hc	*RV* (%)	9 ± 7 (0–21)	9 ± 5 (0–19)	15 ± 12 (1–39)	9 ± 7 (1–22)	9 ± 7 (1–22)	12 ± 12 (0–38)
*K* (%)	83 ± 3 (79–88)	79 ± 7 (55–88)	79 ± 5 (68–86)	85 ± 6 (66–90)	85 ± 4 (78–90)	81 ± 12 (39–89)
*FP* (%)	14 ± 5 (7–26)	16 ± 8 (6–38)	15 ± 7 (8–33)	12 ± 7 (5–28)	11 ± 5 (5–23)	18 ± 10 (10–50)
*FN* (%)	15 ± 5 (9–24)	18 ± 4 (12–24)	20 ± 9 (8–39)	15 ± 6 (8–26)	15 ± 6 (8–26)	12 ± 6.3 (6–26)
*MIV* (%)	2.1 ± 1.6 (0–4.8)	1 ± 1 (0–2.7)	3.1 ± 4.7 (0–13)	0.8 ± 0.9 (0–2.7)	0.8 ± 0.9 (0–2.7)	1.1 ± 1.1 (0–3.1)
*Dm* (mm)	0.6 ± 0.2 (0.3–0.9)	0.7 ± 0.6 (0.4–.8)	0.9 ± 0.4 (0.4–2.2)	0.6 ± 0.4 (0.3–2.1)	0.5 ± 0.2 (0.3–.9)	0.8 ± 1 (0.3–4.4)
*DM* (mm)	4.4 ± 1 (3–7.5)	4.3 ± 2.2 (1.5–12)	6.4 ± 2.5 (3.3–12)	4.4 ± 2.2 (2.4–12)	4 ± 1.1 (2.4–6.7)	4.7 ± 3.3 (3–16)
Am	*RV* (%)	12 ± 9 (1–36)	9 ± 7 (2–25)	26 ± 24 (3–81)	10 ± 9 (0–31)	10 ± 9 (0–31)	12 ± 9 (2–36)
*K* (%)	73 ± 5 (65–81)	83 ± 3 (76–87)	62 ± 10 (45–78)	77 ± 3 (71–82)	77 ± 3 (71–82)	77 ± 2 (74–81)
*FP* (%)	23 ± 7 (12–38)	17 ± 5 (8–26)	22 ± 7 (9–34)	17 ± 4 (10–29)	17 ± 4 (10–29)	23 ± 5 (16–33)
*FN* (%)	19 ± 6 (10–29)	13 ± 4 (5–19)	32 ± 16 (14–62)	20 ± 7 (8–34)	20 ± 7 (8–34)	14 ± 4 (4–21)
*MIV* (%)	0.4 ± 0.5 (0–1.7)	2.8 ± 2.9 (0–7.6)	0.3 ± 0.4 (0–1.4)	0.5 ± 0.7 (0–2.4)	0.5 ± 0.7 (0–2.4)	1.7 ± 3 (0–11)
*Dm* (mm)	1 ± 0.2 (0.6–1.4)	0.5 ± 0.1 (0.4–0.7)	1.5 ± 0.6 (0.8–2.7)	0.8 ± 0.2 (0.5–1.2)	0.8 ± 0.2 (0.5–1.2)	0.8 ± 0.2 (0.6–1.1)
*DM* (mm)	5.1 ± 1.1 (3.6–7.1)	2.8 ± 0.6 (2.2–4.3)	6.2 ± 1.3 (4.3–9.4)	4.1 ± 0.9 (2.9–5.7)	4.1 ± 0.9 (2.9–5.7)	4 ± 0.7 (3–5.6)

Values = average ± standard-deviation (minimum – maximum).

## Appendix A Atlas mismatch detection and correction

The four steps of the mismatch detection and correction strategy are all related. The order in which they were applied was chosen to be from the more global mismatch to the more local one, ending with a last check on the global atlas once all the corrections are done.

### Detection and correction of global misplacement

The first test detected whether global atlas misalignment had taken place in the region of interest. It was based on the assumption that Hc^0.5^ misalignment would correspond to a wider intensity range. σ_Hc_^0.5^, the standard deviation of the intensity on Hc^0.5^, was therefore compared to *σ*_Hc_^0.5*E*^, the standard deviation on Hc^0.5*E*^, created by eroding Hc^0.5^ with a 1-voxel element. For large misplacements of Hc^0.5^, Hc^0.5*E*^ would also include comparable proportions of extra-hippocampal tissues, and the ratio *R*_global_ = σ^0.5*E*^/(σ^0.5^ − σ^0.5*E*^) would be above the threshold, defined as 4 standard deviations above the average. If misplacement was detected, it was corrected by iteratively minimising *R*_*global*_ through a search amongst the objects Hc^0.5^ translated by one voxel in the 6 main orientations (*x* ± 1, *y* ± 1, *z* ± 1).

### Detection and correction of mis-estimated atrophy

The second mismatch test was aimed at detecting cases in which the deformed atlas failed to match the Hc atrophy. ${\tilde{i}}^{0.5}$, the average intensity on Hc^0.5^, was compared to ${\tilde{i}}^{GM}$, the average intensity of *GM* on *BB*_HcAm_; the assumption was that an object Hc^0.5^ which was overestimated compared to the actual Hc would include too many cerebrospinal fluid (dark) voxels, and the ratio $R_{\text{atrophy}}={\tilde{i}}^{0.5}/{\tilde{i}}^{GM}$ would be too low; mismatch was detected when *R*_atrophy_ was under the threshold, defined as 4 standard deviations under the average. The correction relied on shrinking *IPA*_Hc_ by erosion, to create *IPA*^*E*^_Hc_. To do so, the atlas was divided into probability level objects, *PL*, as follows:

(7) $$PL_{\text{Hc}}^{i}=\left\{v,IPA_{\text{Hc}}\left(v\right)\geq \frac{i}{N}\right\},i=1\ldots N\text{.}$$

The *PL* objects were eroded with a 1-voxel element to create *PL*^*Ei*^_Hc_. The eroded atlas *IPA*^*E*^_Hc_ was defined as follows:

(8) $$IPA_{\text{Hc}}^{E}\left(v\right)=\frac{1}{N}\sum_{i=1}^{N}\delta_{PL_{\text{Hc}}^{E^{i}}}\left(v\right)\text{,}$$

δ_*O*_ being the characteristic function of *O* (if *v*∈*O*, δ_*O*_(*v*) = 1, else δ_*O*_(*v*) = 0). *IPA*^*E*^_Hc_ then replaced *IPA*_Hc_. The correction was taken into account only if the initial object created from *IPA*^*E*^_Hc_ was not below 5% of the average size.

### Detection and correction of initial object misplacement

The third mismatch test detected a misplacement of Hc^init^. It compared σ^init^, the standard deviation of the intensity on Hc^init^, to σ^GM^, the standard deviation of the intensity of *GM* estimated on *BB*_HcAm_. The assumption was that, if Hc^init^ was not entirely included in the target Hc, it would include a wider range of intensities, and the ratio *R*_init_ = σ^init^/σ^*GM*^ would be too high, the threshold being defined as 4 standard deviations above the average. Initial misplacement was corrected as global misplacement, the whole atlas *IPA*_Hc_ being then translated according to the displacement of the initial object.

### Final detection of global misplacement

It could happen that Hc was in fact not misplaced but mis-rotated, and would not be corrected by the previous quick strategy. This test was the same as in the section Detection and correction of global misplacement. In the case that no better position had been found during the correction process, misplacement was detected again and the probabilistic constraint was removed for Hc, thus resulting in a segmentation not wrongly biased by the probabilistic atlas.

### Illustration of the measures of mismatch

In order to estimate the actual misplacement of the registered atlas and evaluate how well it was detected by our strategy, we computed quantitative indices for comparing atlas-derived objects and manual segmentation in our three test groups. Note that the misplacement detection does not require manual segmentations; these are used to facilitate visualisation. This figure also shows how the threshold of 4 standard deviations divides between the subjects with and without actual mismatch, and that the atlas is correctly registered in most subjects. The threshold was chosen at 4 instead of 2 standard deviations, in order to detect only large mismatches.

For the global misplacement of the registered atlas, we compute the average symmetric distance between the 0.5-level object and the manual segmentation; it is displayed as a function of the test value in Fig. 5a. Five global misplacements are correctly detected: three for sclerotic hippocampi, one for a TLE patient with grey matter heterotopia in the hippocampal region, and a normal control in whom the left hippocampus was obviously and largely rotated compared to the standard position.

For the mis-estimation of Hc atrophy by the registration procedure, we compute the relative error on volume (the absolute value is not taken here, as only over-estimations are of interest) between the 0.5-level object and the manual segmentation; it is displayed as a function of the test value in Fig. 5b. A large mis-estimated atrophy is detected for one single sclerotic hippocampus. The registration was correct regarding atrophy estimation in the other sclerotic hippocampi.

Finally, in order to detect the actual initial object misplacement, we can compute the false positive ratio between the initial object and the manual segmentation, thus indicating the proportion of misclassified voxels in the initial object; it is displayed as a function of the test value in Fig. 5c. The largest initial object misplacement was not detected, because the initial Hc was partly in Am, which intensity is nearly similar as Hc's intensity.

## Appendix B Atlas constraints

To introduce the atlas constraint was to consider four discrete probability zones *PZ*:

(9) $$\begin{array}{l} PZ^{0}=\left[v\in \Omega ,IPA_{O}\left(v\right)=0\right]\\ PZ^{1}=\left[v\in \Omega ,IPA_{O}\left(v\right)=1\right]\\ PZ^{1-T}=\left[v\in \Omega ,1-T\leq IPA_{O}\left(v\right)<1\right]\\ PZ^{T}=\left[v\in \Omega ,0<IPA_{O}\left(v\right)\leq T\right] \end{array}$$

*T* being here equal to 0.25, *PZ*^0^ and *PZ*^0.25^ corresponded to low likelihood zones; *PZ*^1^ and *PZ*^0.75^ corresponded to high likelihood zones. In order to evaluate the usefulness of each region, four constraints C1, C2, C3 and C4 were built including more and more regions, C1 using the information in *PZ*^0^, C2 in *PZ*^0^ and *PZ*^1^, C3 in *PZ*^0^, *PZ*^1^ and *PZ*^0.75^, and C4 in the four *PZ*s.

The choice of the four probability thresholds defining the probability zones *PZ* was motivated by the “U” shape of the probability histogram and the true (*TP*) and false positives (*FP*) rates as a function of *IPA*_*O*_(*v*) for the leave-one-out atlases (Figs. 6a and b). The choice of the probabilities 0 and 1 is natural; the intermediate levels reflect the plateaus observed in the true positive rate below the 0.75-probability level, respectively in the false positive rate above the 0.25-probability level, indicating that the number of voxels correctly (respectively incorrectly) labelled in the atlas increases in both structure dramatically above (respectively below) the indicated probability threshold.

The γ*_O_^PZ^* values were empirically chosen, with respect to the γ*_O_^AZ^* values and after a preliminary test on S1–S16, and were taken as follows (for probability zones not taken into account here, γ*_O_^PZ^* = 1):

(10) $$\left\{ \begin{array}{l} v\in PZ^{0}\Rightarrow \gamma_{O}^{PZ}\left(v\right)=0.75\\ v\in PZ^{0.25}\Rightarrow \gamma_{O}^{PZ}\left(v\right)=0.9\\ v\in PZ^{0.75}\Rightarrow \gamma_{O}^{PZ}\left(v\right)=1.5\\ v\in PZ^{1}\Rightarrow \gamma_{O}^{PZ}\left(v\right)=2 \end{array}\right.\text{.}$$

The *PZ* and γ*_O_**^PZ^* values are illustrated in Fig. 6c and d for the 4 levels of constraint evaluated here.

The effect of the constraint can also be characterised by comparing the results obtained when adding the *PZ* regions sequentially, for the atlas construction cohort. The results are shown in Table 5 for a subset of four quantitative indices. They show that the refinement of the constraint tends to improve the segmentation accuracy. The difference between C3 and C4 is slight, C4 being mostly better for Am, and thus considered as the optimal constraint.

The influence on the segmentation results when varying the threshold *T* between 0.1 and 0.5 is very low. When *T* varies between 0.1 and 0.3, *RV* and *K* vary less than 1 percentage point for Hc and Am, apart from *RV* for Am which varies of ∼ 3 percentage points for C3 and 1 percentage point for C4. When *T* varies between 0.1 and 0.5, the variation is still less than 1 percentage point for *K*, for Hc and Am; it becomes larger for *RV* for Am with C3 (∼ 6 percentage points), but remains low enough for Hc (∼ 2 percentage points) and for both structures with C4 (< 1 percentage point for Hc and ∼ 2 percentage points for Am). The variation in the values of the quantitative indices is much larger when switching between C1 and C4 than when varying *T*.

The effect of varying γ*_O_^PZ^* values has also been studied. For each *PZ*, we have run the segmentation with the constraint in which this *PZ* is introduced (for example C2 for *PZ*^1^) while letting the value of γ*_O_^PZ^* vary of 30% of the parameter value, while ensuring the constraint that unlikely zones will correspond to values lower than 1 and vice-versa. Results for *RV* and *K* values for Hc and Am are presented in Fig. 6e.

- For *PZ*^0^, γ*_O_^PZ^* varies between 0.5 and 1, using C1; *RV* varies of 3 percentage points for Hc and 2 percentage points for Am and *K* varies of 1 percentage point for Hc and 5 percentage points for Am.
- For *PZ*^1^, γ*_O_^PZ^* varies between 1.4 and 2.6, using C2; *RV* and *K* for Hc and Am vary less than 1 percentage point.
- For *PZ*^0.75^, γ*_O_^PZ^* varies between 1 and 2 using C3; *RV* for Hc and *K* for Hc and Am vary between 1 and 2 percentage points; *RV* for Am varies of about 3 percentage points.
- For *PZ*^0.25^, γ*_O_^PZ^* varies between 0.6 and 1, using C4; *K* varies of about 3 percentage points for Hc and 1 percentage point for Am, and *RV* varies of 12 percentage points for Hc and 7 percentage points for Am. Nevertheless, as γ*_O_^PZ^* is set to 0.75, and as the confidence in *PZ*^0.25^ is lower than the confidence in *PZ*^0^, the only real range which has a meaning for γ*_O_^PZ^* is 0.75 to 1; in this range, *K* varies of about 1 percentage point for Hc and Am, and *RV* varies of 6 percentage points for Hc and 3 percentage points for Am.

These variations are thus very small compared to the variation range of the parameters; only γ*_O_^PZ^* associated with *PZ*^0.25^ causes a larger variation of *RV* for both structures. First, the variation of the indices is to be compared to the variation between with the values of the indices with and without the atlas constraint: 2 percentage points for *RV* for Hc, 4 percentage points for *K* for Hc, 6 percentage points for *RV* for Am, 8 percentage points for *K* for Am. This variation is explained by adding the constraints in the 4 *PZ*s at the same time; the values reported in Table 5 when adding the *PZ*s one by one are consistent with the variation range reported above, apart from *PZ*^0.25^, for which the improvement (between C3 and C4) is less obvious.

## Appendix C Influence of the probabilistic atlas on the segmentation results

The influence of the probabilistic atlas introduced in the segmentation was tested in two ways, for S1–S16. First, the influence of the number of subjects used to build the atlas was evaluated, through a “learning curve” procedure, by comparing the results of the segmentation with an atlas built from 4, 8, 12 subjects, in addition to the leave-one-out procedure and the full atlas built from S1–S16. Second, the influence of the age range and the manual segmentation protocol on the final result was established by using an atlas with the 10 adults from the 18 subjects IBSR database (the MR brain data sets and their manual segmentations were provided by the Center for Morphometric Analysis at Massachusetts General Hospital and are available at http://www.cma.mgh.harvard.edu/ibsr/), using the manual segmentations supplied. All tests were performed without change in the segmentation parameters.

For the “learning curve” strategy, apart from the full atlas, the segmented subject was never included when building the atlas used in its segmentation. The first five columns of Table 6 show the evolution of the values of the quantitative indices for S1–S16 when increasing the size of the atlas sample, for the automatic segmentation with atlas constraint (a), and the 0.5-probability object (b). For *RV* and *K*, the effect on the mean values is noticeable for the 0.5-level object but far more reduced for atlas-constrained automatic segmentation. For *DM*, the effect is weak for both the segmentation and the 0.5-level object, on both mean and maximal results. Finally, it can be noted that the differences between the leave-one-out atlas and the full atlas are very small. Therefore, for the automatic segmentation, a sample of scans from 15 healthy subjects is likely to be sufficient to take into account the variability of the young healthy subjects within our framework.

For the test with the atlas built from IBSR subjects, the results are reported in the last column of Table 6. The values for Hc are quite similar to those that were obtained above for the atlas built with 8 and 12 subjects. These results show that the region deformation step driven by anatomical prior based on our manual segmentation protocol is little influenced by the probabilistic atlas for Hc. For Am, the results are not as good, showing a larger influence of the atlas on the results; Am is not as well defined as Hc, and the influence of the atlas constraint in the segmentation process is larger. The difference between the two probabilistic atlases can be inferred from the 0.5-level objects, which show that the one derived from the IBSR10 would be worse compared even to the 4 subject atlas. This demonstrates that while atlas prior information is different, the segmentation process is able to correct for the differences.

## Appendix D Influence of intensity and initialisation parameter settings

The predefined ratios are used to determine the intensity parameters used in the data attachment terms. The influence of their setting on the segmentation results has been evaluated for S1–S16, as displayed in Figs. 7a and b. The ratios for mean intensity and tolerance were varied by ± 10% first for Hc (Fig. 7a) and then for Am (Fig. 7b). There are large areas of stability for both sets of parameters. Outside of these areas, the variation affects *RV* (range for Hc when varying Hc parameters: ∼ 6% to 15%) more strongly than *K* (range for Hc when varying Hc parameters: ∼ 83% to 88%).

The threshold in the probability map and the size of the structuring element used to create the initial object have also been varied (initial probability between 0.5 and 1, erosion structuring element between 0 and 2 mm), and the results are displayed in Fig. 7c. They show that a large variability of initialisation parameters will induce only a small variability in the results (less than 1 percentage point for K, less than 2 percentage points for *RV*), while the size of the initial objects varies between 0 and 2.5 cm^3^ for Hc and 0 and 1.3 cm^3^ for Am.
